# Editorial: New immunotherapeutic and pharmacological targets and strategies in haematological malignancies

**DOI:** 10.3389/fphar.2023.1161811

**Published:** 2023-02-20

**Authors:** Mark Thomas Shaw Williams, Yong-Mi Kim, Monica L. Guzman

**Affiliations:** ^1^ Charles Oakley Laboratories, Department of Biological and Biomedical Sciences, Glasgow Caledonian University, Glasgow, United Kingdom; ^2^ Children’s Hospital of Los Angeles, Department of Pediatrics, University of Southern California, Los Angeles, CA, United States; ^3^ Weill Cornell Medicine, Cornell University, White Plains, NY, United States

**Keywords:** haematological malignancies, therapy resistance, microenvironment, immunotherapy, drug repurposing

The treatment landscape of the oncology field in the last decade has been transformed, due to the introduction of new immunotherapeutic and pharmacological strategies, including immune checkpoint inhibitors (ICIs) (Chen et al.). This has led to improved outcomes for solid cancer patients. Despite advances in haematological malignancies, treatments and outcomes for patients however, have not significantly changed. This has been partly driven by the paucity in the introduction of efficacious therapies. Furthermore, therapy resistance significantly contributes towards treatment failure and relapse in blood cancer patients. Consequently, there has been a concerted effort to investigate alternative including natural products (NPs) (Yu et al., Li et al.) and re-purposed therapeutic approaches, to circumvent therapy resistance and induce lasting remission in blood cancer patients.

This Research Topic is aimed at collating articles appropriate to enhance our awareness/understanding of the pre-clinical and clinical efficacy of, novel, existing and alternative therapeutic approaches, with the goal of improving outcomes in blood cancer patients.

In this special e-Research Topic there are four articles covering the aforementioned aspects.

NPs as effective alternative therapies for the treatment of haematological malignancies has been the most discussed/evaluated aspect. Two out of four articles were associated with this Research Topic, and represent a growing number of studies exploring the therapeutic potential of NPs. Currently, AML patients are treated with toxic traditional drugs including daunorubicin (DNR), that not only target leukemic cells, but also non-cancerous by-stander cells. Although these drugs induce remission in 80%–85% of patients over 60% exhibit disease relapse, and due to a lack of effective salvage treatments, most patients will die (Li et al.). Moreover, relapse can arise due to the persistence of leukemic stem cells (LSCs), which can be enriched by DNR treatment (Wang et al., 2020). In AML there is a need to investigate leukemic cell specific therapeutics, which can eradicate relapse-promoting cancer stem cells (CSCs), to induce long term remission.

Research on alternative pharmacologically active agents like NPs, is highly promising, but still at pre-clinical stages. Excitingly several NPs exhibit anti-leukaemic properties (Kinneer et al., 2017; McGill et al., 2018), Jiyuan ordonin A (JOA) can overcome differentiation blockade in common AML subtypes Li et al. demonstrate that JOA suppressed and prompted the *in vitro* proliferation and differentiation of several AML cell lines respectively, and reduced LSC proliferation *in vivo*. The second article discusses NPs known as traditional Chinese medicinal materials (TCMMs) Yu et al. highlight several bioactive TCCMs, including baicalein, which exhibit anti-multiple myeloma (MM) activity. These bioactive components exert anti-proliferative, pro-apoptotic, and chemosensitising effects on MM cells, by inhibiting key signalling pathways. Interestingly baicalein, could have wider therapeutic benefit, potentially exhibiting anti-CSC activity not only in the context MM, but also AML. This could occur *via* baicaleins’ ability to inhibit a drug efflux transporter/pump expressed by side population cells; enriched in LSCs (Moshaver et al., 2008) and MM stem cells (MMSCs) (Gao et al., 2016), and potentially mediates chemoresistance (Raaijmakers et al., 2005).

Although new drug approvals for haematological malignancies, have been limited drug re-purposing offers a faster and more cost effective alternative for introducing new pharmacological strategies.

Standard drug development processes, cost $2 billion and required 14 years in 2022 to take a new drug from benchside to bed/clinic (https://ftloscience.com/process-costs-drug-development/), drug re-purposing can reduce the time to clinic by two-fold (average 6 years), and has the added benefit of reducing severe adverse effects, as existing therapies have been implemented in other diseases (Nosengo, 2016). The anti-helminth/worm agent mebendazole (MBZ), has been assessed for its therapeutic potential in Chronic Myeloid Leukaemia (CML) (Daniel et al.). CML treatment was revolutionised by the introduction of tyrosine kinase inhibitors (TKIs). However, TKI resistance can develop due to mutations in abl kinase (TKI target), this includes the T315I gate-keeper mutation. This prompted the development of second and third generation of TKIs, including dasatinib and ponatinib. Studies by Daniel et al., demonstrate that unlike clinical TKIs, MBZ binds with high affinity to an allosteric site on the abl kinase, and could be potentially effective in CML patients carrying the T315I mutation. Furthermore, when combined with imagines and dasatanib MBZ induced synergistic cell killing in chemosensitivity and chemoresistant CML cells.

The fourth paper in the series, reviews several pre-clinical and clinical studies/trials evaluating the expression of well-established IC proteins [e.g., programmed cell death protein 1 (PD-1)], and more recently identified/studied IC proteins [e.g., T-cell immunoglobulin and mucin domain-containing protein-3 (TIM-3)], in the context of common subtypes of peripheral t-cell lymphoma (PTCLs) (Chen et al.). Several ICIs are available for treating solid cancers. However, only two ICIs have been approved for the treatment of haematological malignancies. This is multi-factorial, including the observation that cancers with a low tumour mutational burden (TMB) (e.g., AML), are likely non-responsive/resistant to ICI therapy (Klempner et al., 2020). ICIs are thought to be efficacious in patients with a high TMB, as the response involves the induction of anti-cancer immune responses, in which neoantigens are expressed in tumours rather than in non-cancerous/healthy cells. In PTCL, TMB varies considerably based on subtype (Heavican et al., 2019), and that specific subtypes, including PTCL-NOS patients with a *p53* mutation, exhibit a higher TMB as compared *p53* wild-type patients. Interestingly, in AML patient’s *p53* mutational status correlates with higher TMB and immune signalling (Wen et al., 2021). Consequently, one could speculate that *p53*, could serve as a biomarker to predict patients across several haematological malignancies that may respond to ICIs.

In summary, conclusions of the above studies and reviews signify new relevant data on alternative and re-purposed therapeutic strategies, exhibiting the potential to tackle unmet clinical obstacles, including therapy resistance in blood cancer patients. Despite the vast evidence on this highly important Research Topic, these published articles demonstrate that there are still numerous facets to be elucidated in the captivating area of new targets and therapies in haematological malignancies. Following the reading of this Research Topic, certain themes as, pre-clinical and clinical efficacy of NPs, and the potential of drug repurposing, will appear more apparent to the reader, strengthening the idea that studies identifying and targeting new mechanisms in haematological malignancies, *via* new and existing drugs, are still critically required to improve clinical outcomes in blood cancer patients.
